# Bronchoalveolar lavage/blood ratio of surface TREM-1 on CD14-positive monocytes is diagnostic of ventilator-associated pneumonia

**DOI:** 10.1186/cc9701

**Published:** 2011-03-11

**Authors:** V Grover, P Kelleher, D Henderson, P Pantelidis, F Gotch, N Soni, S Singh

**Affiliations:** 1 Chelsea and Westminster NHS Foundation Trust, London, UK; 2 Imperial College Healthcare NHS Trust, London, UK; 3 Imperial College, London, UK

## Introduction

Biomarkers offer the possibility to speed up diagnosis of ventilator-associated pneumonia (VAP) and differentiate it from nonpulmonary infection. One such marker, the triggering receptor expressed on myeloid cells-1 (TREM-1), exists as a soluble protein and a surface receptor expressed on monocytes and neutrophils [1]. The purpose of the study was to determine the diagnostic utility of surface TREM-1 levels in VAP.

## Methods

Paired bronchoalveolar lavage (BAL) and blood were obtained from 25 VAP patients, 15 ventilated non-infected controls, 10 ventilated patients with nonpulmonary infection and 25 nonventilated controls. VAP diagnosis was by clinical pulmonary infection score (CPIS) and semiquantitative microbiology. BAL and blood monocytic and neutrophilic levels of surface TREM-1 and CD11b (leukocyte activation marker) were assessed using flow cytometry. Monocytes were CD14-positive. Soluble TREM-1, IL-1β, IL-6 and IL-8 were measured using ELISA. BAL dilution was corrected by urea assay.

## Results

**Figure 1:**
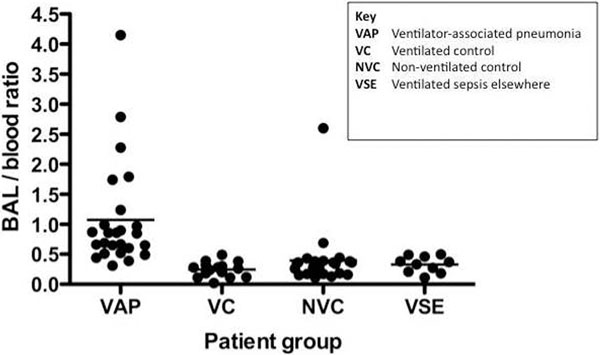
**BAL/blood monocytic TREM-1 ratio**.

See Figure 1. The BAL level of monocytic surface TREM-1 was elevated in VAP. For ventilated patients, the area under the ROC curve (AUC) was 0.87 for diagnosing VAP, with sensitivity 72% and specificity 80%. Blood levels did not differ between the groups. However, the BAL/blood ratio improved diagnostic accuracy further. The AUC was 0.97, sensitivity 84%, specificity 92% and positive likelihood ratio 10.5. The ratio differentiated pulmonary from nonpulmonary infection. The BAL/blood ratio of monocytic CD11b was 0.78. The BAL levels of neutrophil surface TREM-1, soluble TREM-1, IL-1β and IL-8 had AUCs of 0.75, 0.76, 0.81 and 0.85, respectively.

## Conclusions

The BAL/blood ratio of monocytic surface TREM-1 diagnoses VAP and differentiates pulmonary from nonpulmonary infection. CD14 and TREM-1 may have a role in the pathogenesis of VAP.
